# Supplementary material for: Absence of association between host genetic mutations in the ORAI1 gene and COVID-19 fatality
Source: PLoS One. 2022 Feb 3;17(2):e0263303. doi: 10.1371/journal.pone.0263303 (PMC8812896; doi:10.1371/journal.pone.0263303)
Supplement: Attachment — Submitted filename: Response to Reviewers_1.docx [file pone.0263303.s001.docx]

Responses to Reviewers

Editor

1. *Please ensure that your manuscript meets PLOS ONE's style requirements,* *including* *those for file naming.*

Response: The manuscript style requirements were reviewed in the manuscript.

2. *In your Data Availability statement, you have not specified where the minimal data set underlying the results described in your manuscript can be found. PLOS defines a study's minimal data set as the underlying data used to reach the conclusions drawn in the manuscript and any additional data required to replicate the reported study findings in their entirety. All PLOS journals require that the minimal data set be made fully available. For more information about our data policy, please see* [*http://journals.plos.org/plosone/s/data-availability*](http://journals.plos.org/plosone/s/data-availability)

Response: Thanks for your comments. The following Data Availability Statement describes where the data set underlying the analysis can be found.

“The data underlying the results presented in the study are available from the UK Biobank on application via the UK Biobank online access management system (<http://www.ukbiobank.ac.uk>).”

3. *Thank you for stating the following in the Acknowledgments Section of your manuscript: “This work was supported by British Heart Foundation fellowships to MAB and HS (FS/18/12/33270, FS/17/66/33480) and Leeds Cardiovascular Endowment support to CWC. This work was conducted using UK Biobank datasets. We thank the UK Biobank participants and staff. This work was undertaken on ARC3, part of the High-Performance Computing (HPC) facilities at the University of Leeds, UK.”*

Response: We appreciate the comments. The funding statement is now removed from the Acknowledgments Section to be only stated in the Funding Section.

Reviewer #1

1. *Study based on a biobank dataset to clarify the role of genetic variants within the ORAI1 gene with positivity and mortality for COVID-19. The study design is not clear, the authors do not accurately describe what the main objective is; these aspects should be better defined.*

Response: We have more clearly defined the rationale for the study, the design and our objective on page 3, line 73-79, in the untracked version of the revised manuscript.

2. *The results demonstrate that there is no significant association between ORAI1 variants and COVID-19 positivity or fatality in the study participants, despite the well-established roles of ORAI1 in immune response and inflammation and the successful inhibition of ORAI1 in clinical studies. Authors should explain the reason for this discrepancy in more detail than they have already done.*

Response: We added the following clarification in the discussion to explain the potential reasons for not finding significant evidence that ORAI1 variants influence the susceptibility to COVID-19 positivity or fatality.

“This absence of genetic association between ORAI1 variants and COVID-19 fatality doesn’t eliminate the possible implication of ORAI1 in disease pathogenesis through altered gene expression levels or channel activity and therefore it does not eliminate the potential usefulness of targeting ORAI1 as a therapeutic target for COVID-19. Our analysis was constrained to participants from White British (Caucasian) background, due to the small number of cases from other ethnic groups in this data set, and therefore possible implication of ORAI1 variants in COVID-19 fatality in different ethnic groups couldn’t be ruled out. Further studies are needed to investigate whether ORAI1 polymorphisms influence the susceptibility to COVID19 positivity or fatality in different ethnic populations. Additionally, the age range of participants was confined to age 50–84 years and didn’t include the younger age group with the lower risk of adverse COVID-19 illness. Because of these geographical, ethnic and age limitations, our finding may not reflect the wider more diverse population.” Page 10-11, Line 199-211 in the untracked version of the revised manuscript.

Reviewer #2

1. *Introduction: in my opinion it should be shortened and focused on the research question.*

Response: We have now shorted the introduction for brevity.

2. *Methods: the frequency of ORAI1 SNP polymorphism should be detailed and considered to estimate a sample large enough to test the research question.*

Response: To evaluate the effect of the variants frequencies in this locus, we included in our analysis the ORAI1 common variants with minor allele frequency more than 5%, as well as the less frequent variants with minor allele frequency more than 1%. We found no significant evidence that either the common (MAF > 5%) or rare (MAF > 1%) variants influence the susceptibility to COVID-19 positivity or fatality. We have added the following remarks to the revised manuscript to highlight this point.

“We did not observe significant association between common variants with MAF > 5% or less frequent variants with MAF > 1% in the ORAI1 gene with COVID-19 positivity or fatality in the examined population.” Page 7, Lines 145-147 in the untracked version of the revised manuscript.

3. *Methods: did the authors collected data regarding SARS-CoV-2 major variants? If so, wouldn’t it be interesting to test whether an association with ORAI1 SNP could be find?*

Response: We appreciate reviewer’s suggestion. Although it is interesting to link ORAI1 SNPs with the major SARS-CoV-2 variants, these data are currently not available in the UK Biobank database. Therefore, studying the association between ORAI1 SNPs in this cohort and SARS-CoV-2 variants is currently not possible. We added the following remarks in the discussion to highlight this limitation in the revised manuscript.

“Our analysis was also limited by the lack of information about the vaccination status of the participants and about the prevalence of the SARS-CoV-2 variants of concern in the examined population.” Page 11, Lines 211-213 in the untracked version of the revised manuscript.

4. *Methods: the authors should report the percentage of vaccinated people.*

Response: This genetic data is approved and provided by Public Health England through UK Biobank. Unfortunately, the vaccination data is yet to be made available to the scientific community. We appreciate this could be a limitation in the study. This limitation is now highlighted in the manuscript. Page 11, Lines 211-213 in the untracked version of the revised manuscript.

5. *Results: it is surprising that COVID-19 fatal and non-fatal case have similar average age, gender distribution and hypertension rate. These data are in contrast with the great majority of results reported in literature and should be discussed in more details.*

Response: it is true that the age, gender and number of cases with hypertension were comparable in the *COVID-19 fatal and non-fatal* groups*.* This could be attributed to the fact that the UK Biobank dataset contains the genetic data for individuals aged between 50 to 84 and only a subset of COVID-19 data was released at the time of analysis. To clarify this and recognise the limitations, we added the following remarks to the revised manuscript.

“We did not observe substantial differences between the mean age of fatal (69.1 ± 8.4 years) and non-fatal (68.8 ± 8.1 years) COVID-19 cases, which could be attributed to the confined age range of the participants between 50 and 84 years. Therefore, the younger age group with a lower risk of serious COVID-19 illness was not captured in our analysis.”. Page 5, Lines 120-124 in the untracked version of the revised manuscript..

“The age range of participants was confined to age 50–84 years and didn’t include the younger age group with the low risk of adverse COVID-19 illness. Because of these geographical, ethnic and age limitations of the examined dataset, our finding may not reflect the wider more diverse population.” Page 11, Lines 208-211 in the untracked version of the revised manuscript.

6. *Results: Data regarding the prevalence of hearth problem in the COVID-19 fatality group should be checked (just 1 over 332?).*

Response: Thanks for your comment. We have noticed a calculation error that attributes to this low number of cases with heart problem. We took the opportunity to correct this error and further examine the prevalence of additional more specific and relevant co-morbidities (Myocardial infarction and Chronic Obstructive pulmonary disease) in the examined population. Table 1 with the basic characteristics of the study participants is now updated. Page 6-7 in the untracked version of the revised manuscript.

**Table 1. Basic characteristics of the study participants**

| **characteristic** | **All** | **COVID-19 Positive** | **COVID-19 Negative** | **COVID-19 Fatalities** | **Non-fatal COVID-19** |
| --- | --- | --- | --- | --- | --- |
| **N*** | **41146** | **7462** | **33684** | **332** | **7130** |
| **Mean age, years ± SD**** | 68.8 ± 8 | 68.8 ± 8.1 | 68.7 ± 8 | 69.1 ± 8.4 | 68.8 ± 8.1 |
| **Gender** | | | | | |
| **Female, n (%)** | 22076 (53.65%) | 3978 (53.3%) | 18098(53.7%) | 181 (54.52%) | 3797 (53.25%) |
| **Male, n (%)** | 19070 (46.35%) | 3484 (46.7%) | 15586 (46.27%) | 151 (45.48%) | 3333 (46.75%) |
| **Co-morbidities** | | | | | |
| **Stroke, n (%)** | 770 (1.87%) | 149 (2.0%) | 621 (1.84%) | 11 (3.31%) | 138 (1.94%) |
| **Hypertension, n (%)** | 12788 (31.08%) | 2018 (27.04%) | 10770 (31.97) | 163 (49.1%) | 1855 (26.02%) |
| **Diabetes, n (%)** | 2520 (6.12%) | 417 (5.59%) | 2103 (6.24%) | 52 (15.66%) | 365 (5.12%) |
| **Asthma, n (%)** | 5562 (13.52%) | 1020 (%) | 4542 (13.48%) | 34 (10.24%) | 986 (13.83%) |
| **Heart/cardiac problem, n (%)** | 210 (0.51%) | 38 (13.67%) | 172 (0.51%) | 4 (1.20%) | 34 (0.48%) |
| **Myocardial infarction, n (%)** | 1364 (3.32%) | 229 (3.07%) | 1135 (3.37%) | 26 (7.83%) | 203 (2.85%) |
| **Chronic Obstructive pulmonary disease, n (%)** | 213 (0.52%) | 39 (0.52%) | 174 (0.52%) | 3 (0.90%) | 36 (0.50%) |

*N, number of individuals, **SD, standard deviation
